# Does aberrant membrane transport contribute to poor outcome in adult acute myeloid leukemia?

**DOI:** 10.3389/fphar.2015.00134

**Published:** 2015-07-02

**Authors:** Alexandre Chigaev

**Affiliations:** Department of Pathology and Cancer Center, University of New Mexico Health Sciences Center, University of New MexicoAlbuquerque, NM, USA

**Keywords:** adult acute myeloid leukemia, poor outcome, gene expression signature, membrane transport, transporters, channels, poor prognosis, prognostic indicators

## Abstract

Acute myeloid leukemia in adults is a highly heterogeneous disease. Gene expression profiling performed using unsupervised algorithms can be used to distinguish specific groups of patients within a large patient cohort. The identified gene expression signatures can offer insights into underlying physiological mechanisms of disease pathogenesis. Here, the analysis of several related gene expression clusters associated with poor outcome, worst overall survival and highest rates of resistant disease and obtained from the patients at the time of diagnosis or from previously untreated individuals is presented. Surprisingly, these gene clusters appear to be enriched for genes corresponding to proteins involved in transport across membranes (transporters, carriers and channels). Several ideas describing the possible relationship of membrane transport activity and leukemic cell biology, including the “Warburg effect,” the specific role of chloride ion transport, direct “import” of metabolic energy through uptake of creatine phosphate, and modification of the bone marrow niche microenvironment are discussed.

## Introduction

In adults and older patients acute myeloid leukemia (AML), the most common type of adult leukemia in USA, is often present as highly resistant disease (RD) with very poor outcomes (Lang et al., 2005). AML is a highly heterogeneous disease, and with the exception of a relatively well defined acute promyelocytic leukemia (APL) characterized by *t*(15;17)(q22;q21) PML-RARA fusion, it exhibits a highly diverse clinical course and requires different treatment modalities (Liersch et al., 2014). Consequently, for an informed therapeutic decision a large number of prognostic factors including but not limited to age, white blood cell (WBC) count, the presence of recurring cytogenetic abnormalities, and individual gene mutations needs to be assessed (Liersch et al., 2014). However, a large number of AML cases have risk-indeterminate or normal karyotypes (Cagnetta et al., 2014), and a fewer number of mutations has been identified in most AML genomes than in most other adult cancers (The Cancer Genome Atlas Research Network, 2013; Grove and Vassiliou, 2014). Moreover, epigenetic deregulation can be also a significant part of AML pathogenesis (Larsson et al., 2013). Thus, prognostication of outcomes and residual disease, as well as of clinical course or relapse rates (RRs) poses significant challenges.

Developed in the last decade, global gene expression analysis is now envisioned as an independent prognostic marker that can be used to classify patients into pathologic groups, significantly improving cytogenetic-based classification schemes. As a result, this approach led to the identification of a web of deregulated genes, non-coding RNAs, and epigenetic modifications specifically important for patients with normal cytogenetic evaluations (Cagnetta et al., 2014). However, gene expression profiling can also identify intrinsic biological groups of AML within a well-characterized patient cohort, and thus, have the ability to offer insights into physiological mechanisms of leukemia pathogenesis (Wilson et al., 2006), provided that unsupervised approaches unrelated to other external factors such as survival, clinical signs, karyotypic abnormalities or individual gene mutations are used. Despite the fact that gene expression is typically assessed on the level of mRNA (cDNA or cRNA), it is usually assumed that up-regulated mRNA levels are related to protein expression, and this will lead to a somewhat enhanced protein-specific function.

Here, the analysis of several related gene expression clusters [Cluster B in referenc Wilson et al. (2006) and Clusters 7 and 8 in reference Valk et al. (2004)] identified using unsupervised algorithms and originating from pretreatment patient samples with primary AML (Valk et al., 2004), or previously untreated *de novo* or secondary AML (Wilson et al., 2006) is presented. These clusters appear to be enriched for genes corresponding to proteins involved into transport across cell membranes (transporters, carriers, and channels). Because these clusters are associated with poor outcome, a specific role for membrane transport in adult AML is postulated. Several hypotheses describing a relationship of membrane transport activity and leukemic cell biology are discussed.

## Cluster Analysis and Unsupervised Algorithms

Gene expression signatures are generated using gene-based clustering, which identifies sets of genes that have high probability of being co-expressed (Do and Choi, 2008). Numerous clustering algorithms have been developed, and several excellent reviews that describe clustering approaches have been recently published (Nugent and Meila, 2010; Wang et al., 2013). However, for the purpose of this report it is necessary to describe the two major classes of clustering methodology: supervised and unsupervised (Do and Choi, 2008).

Supervised methods take into account known in advance phenotypic parameters of the analyzed samples, or other descriptors that characterize groups of samples. As a result, the subsets of genes whose level of expression exhibit correlation with the specified class descriptors are identified. The weakness of this method is that it leads to the identification of small gene subsets, often identified as “informative genes,” and other unrelated genes are perceived as noise (Do and Choi, 2008). Therefore, clustering algorithms that employ supervised methods are only as good as the predefined set of class descriptors. For heterogeneous diseases, such as AML, a set of informative genes can be largely dependent on the heterogeneity of the initial data.

For unsupervised approaches the analysis is based on the assumption that the variability of a group of variables depends upon a certain factor (principal component) that is hidden from the observer. This type of analysis can lead to the identification of an underlying pattern in the data, and provide additional clues into a process or biological meaning. This type of analysis is vital when the overall goal is to uncover a previously undefined relationship or to formulate a new hypothesis. However, if a pattern is absent, the results would have no meaning.

## Patients with Worst Overall Survival (OS) and Highest Rates of Resistant Disease

One of the requirements for the analysis of a cohort of patients is the ability to analyze previously untreated *de novo* or secondary AML. The power of a retrospective analysis is that gene expression profiles can be directly correlated to the disease outcomes. It is understandable why a specific focus has been on clusters of genes that correlated with the worst disease-free and overall patient survival (DFS and OS), and high rates of RD. Therefore, cluster B from the report (Wilson et al., 2006), which coincided with the poorest clinical outcomes, an exceptionally high rate (77%) of RD and the lowest DFS, attracted immediate attention. Moreover, the number of significant cluster-defining genes from cluster B from this study was similar to the gene signature from clusters 7 and 8 from (Valk et al., 2004). The majority of the top 50 ranked genes from cluster B (∼90%) represented up-regulated genes, whereas a significant portion of top ranked genes in other clusters were down-regulated (Wilson et al., 2006). Unfortunately, no survival data for clusters 7 and 8 were provided in (Valk et al., 2004).

According to Wilson et al. (2006) cluster B represented an “interesting group of 22 patients,” where the majority (77%) was unresponsive to the induction therapy, and three patients with achieved remission relapsed within the first 16 months. Also, 42% of all cases in cluster B showed normal karyotype, one patient had both *NPM1* and *FLT3* internal tandem duplication (ITD) mutations and two individuals had *FLT3*-ITD (Wilson et al., 2006). In Valk et al. (2004) clusters 7 and 8, where gene signatures were similar to cluster B, the number of cases showing normal karyotype were 67 and 31% respectively, with 22% of cases exhibiting *FLT3*-ITD and 11% with *EVII* overexpression in cluster 7 and 8% of N-*Ras* in cluster 8. Thus, a significant fraction of cases had no recurring cytogenetic abnormalities previously described individual gene mutations.

## Unsupervised Gene Clusters from Two Independent Studies are Enriched for Genes Corresponding to Proteins Involved in Membrane Transport

Previously, Wilson et al. (2006) reported that Clusters 7 and 8 of Valk et al. (2004) had a gene expression profile most similar to cluster B. It has been noted that one of genes overexpressed by these patients was *ABCG2*, a member of ATP-binding cassette (ABC) superfamily of membrane transporters, also termed breast cancer resistance protein (BCRP1), later dubbed as a stem cell marker and a target in cancer stem cell therapy (Ding et al., 2010). Moreover, *ABCG2* protein overexpression in concurrence with *FLT3*-ITD mutation identifies a subgroup of AML patients with significantly worse prognosis (Tiribelli et al., 2011).

Our analysis of the top most significant discriminating genes from all three clusters revealed an unanticipated phenomenon: these clusters were enriched in genes functionally involved in membrane transport – transporters and channels (**Table 1**). Here protein functions are described according to UniprotKB^1^ and Gene Ontology^2^ databases. For gene function annotation from UniprotKB, reviewed (curator-evaluated) computational records (Swiss-Prot) were used. Below, a review of the specific gene functional roles is presented. This review is only intended to show data that are relevant for this particular context. For multiple genes listed below exhaustive literature reviews do exist.

**Table 1:** Genes corresponding to proteins involved into membrane transport from Cluster B in reference Wilson et al. (2006) and Clusters 7 and 8 in reference Valk et al. (2004).

Uniprot Accesion number and link to the original record	Gene names	Protein Names according to UniProt	Protein Function according to UnirpotKB and Gene Ontology	Clusters where can be found	Reported role in cancer/leukemia (see text for details and references)
Q9UNQ0	ABCG2*, ABCP, BCRP, BCRP1, MXR*	ATP-binding cassette sub-family G member 2 (Breast cancer resistance protein; CDw338; Mitoxantrone resistance-associated protein; Placenta-specific ATP-binding cassette transporter; Urate exporter; CD antigen CD338)	High-capacity urate exporter, mediates the export of protoporhyrin IX (PPIX) both from mitochondria to cytosol and from cytosol to extracellular space. Appears to play a major role in the multidrug resistance (MDR) phenotype of several cancer cell lines. Implicated in the eﬄux of numerous drugs and xenobiotics	Cluster B and cluster 8	High expression of ABCG2 mRNA is shown to be an independent prognostic factor for relapse rate (RR) and disease-free survival in adult AML. ABCG2 null alleles define the Jr(a-) blood group phenotype
P14415	*ATP1B2*	Sodium/potassium-transporting ATPase subunit beta-2 [Adhesion molecule in glia (AMOG; Sodium/potassium-dependent ATPase subunit beta-2]	This is the non-catalytic component of the active enzyme, which catalyzes the hydrolysis of ATP coupled with the exchange of Na^+^ and K^+^ ions across the plasma membrane. The exact function of the beta-2 subunit is not known.	Cluster B	No role in leukemia has been reported, However, ATP1B2 down-modulates brain tumor-initiating cell invasion. Epigenetic regulation of expression was reported in malignant gliomas
P51790	*CLCN3*	H(+)/Cl(-) exchange transporter 3 (Chloride channel protein 3; ClC-3; Chloride transporter ClC-3)	Mediates the exchange of chloride ions against protons. Functions as antiporter and contributes to the acidification of the endosome and synaptic vesicle lumen, and may thereby affect vesicle trafficking and exocytosis. Chloride channel activity	Cluster 7	CLC-3 is a regulator of cell cycle in normal and malignant glial cells. Expression of ClC-3 is implicated in cancer drug resistance, and it represents a target of paclitaxel-induced apoptosis
O15247	*CLIC2*	Chloride intracellular channel protein 2 (XAP121)	Can insert into membranes and form chloride ion channels. Voltage-gated chloride channel activity. Modulates the activity of RYR2 and inhibits calcium influx	Cluster B	CLIC2-related chloride intracellular channels CLIC1 and CLIC4, are overexpressed in cancer stem cells and envisioned as novel therapeutic targets. CLIC-like chloride channel is localized at the osteoclast ruﬄed border and together with H^+^-ATPase can acidify bone resorption compartment
Q02094	*RHAG, RH50, SLC42A1*	Ammonium transporter Rh type A (Erythrocyte membrane glycoprotein Rh50; Erythrocyte plasma membrane 50 kDa glycoprotein; Rh50A; Rhesus blood group family type A glycoprotein; Rh family type A glycoprotein; Rh type A glycoprotein; Rhesus blood group-associated ammonia channel; Rhesus blood group-associated glycoprotein; CD antigen CD241)	Rhesus glycoproteins mediate ammonium transport. A part of an oligomeric complex which is likely to have a transport or channel function	Cluster 7	For Rh glycoproteins no direct relation to cancer or cancer cell metabolism has been previously reported. However, rhesus blood group family proteins are important for transport of ammonia, ammonium ion, possibly carbon dioxide, and regulation of acid-base tramsport across membranes
P18577	*RHCE, RHC, RHE*	Blood group Rh(CE) polypeptide (Rh polypeptide 1; RhPI; Rh30A; RhIXB; Rhesus C/E antigens; CD antigen CD240CE)	Ammonia transporter channel (amt) family. A part of an oligomeric complex which is likely to have a transport or channel function	Cluster B and cluster 8	See description for RHAG (above)
Q02161	*RHD*	Blood group Rh(D) polypeptide (RHXIII; Rh polypeptide 2; RhPII; Rhesus D antigen; CD antigen CD240D)	Ammonium transmembrane transporter activity. A part of an oligomeric complex which is likely to have a transport or channel function	Cluster B, clusters 7 and 8	See description for RHAG (above)
P11166	*SLC2A1, GLUT1*	Solute carrier (SLC) family 2, facilitated glucose transporter member 1 (Glucose transporter type 1, erythrocyte/brain; GLUT-1; HepG2 glucose transporter)	Facilitative glucose transporter. This isoform may be responsible for constitutive or basal glucose uptake. Has a very broad substrate specificity; can transport a wide range of aldoses including both pentoses and hexoses.	Cluster B and cluster 7	GLUT1 overexpression is a prognostic indicator for cancer. High levels of GLUT1 is associated with poor responsiveness to chemotherapy in AML. In the presence of several glucose transporters B-ALL cells rely specifically upon GLUT1 to maintain metabolism
P02730	*SLC4A1, AE1, DI, EPB3*	Solute carrier family 4, anion exchanger, member 1 (erythrocyte membrane protein band 3, Diego blood group), AE1, band 3 anion transport protein	Transporter that mediates the 1:1 exchange of inorganic anions. Mediates chloride-bicarbonate exchange in the kidney.	Cluster B	No specific role for AE1 in hematological malignancies can be found. A series of reports describes the role of AE1 in gastric cancer.
P48029	*SLC6A8*	Sodium- and chloride-dependent creatine transporter 1 (CT1; Creatine transporter 1; SLC family 6 member 8)	Creatine transmembrane transport activity, creatine:sodium/chloride symporter activity. Reported to import phosphocreatine, a compound that has high phosphoryl potential and can serve as a source of metabolic energy	Cluster 7	CT1 is highly expressed in tissues/organs that require high energy utilization. This might indicate high energy use in AML cells. SLC6A8 transporter is implicated in the “import” of metabolic energy in the form of phosphocreatine into colon cancer cells
P48067	*SLC6A9*	Sodium- and chloride-dependent glycine transporter 1 (GlyT-1; GlyT1; SLC family 6 member 9)	Glycine:sodium symporter activity. Terminates the action of glycine by its high affinity sodium-dependent reuptake into presynaptic terminals. May play a role in regulation of glycine levels in NMDA receptor-mediated neurotransmission	Cluster 7	GlyT1 transporters are implicated in regulation of pain, and transporter inhibitors are envisioned as drugs for neuropathic pain management for example in bone cancer
Q13228	*SELENBP1, SBP*	Selenium-binding protein 1 (56 kDa selenium-binding protein; SBP56; SP56)	Selenium-binding protein which may be involved in the sensing of reactive xenobiotics in the cytoplasm. May be involved in intra-Golgi protein transport (By similarity). Protein transport	Clusters 7 and 8	SBP1 described as a prognostic indicator of clinical outcome and a tumor suppressor gene (TSG) in multiple cancer types
Q9P0U1	*TOMM7, TOM7, TOMM07, AD-014*	Mitochondrial import receptor subunit TOM7 homolog (Translocase of outer membrane 7 kDa subunit homolog)	Required for assembly and stability of the translocase of the outer membrane (TOM) complex. Protein transmembrane transporter activity. Protein import into mitochondrial matrix	Cluster B	No known association between cancer and TOM7 exists. However, several components of mitochondrial import machinery are reported to be up-regulated in cancer and in some cases can be associated with poor prognosis
P51811	*XK, XKR1, XRG1*	Membrane transport protein XK (Kell complex 37 kDa component; Kx antigen; XK-related protein 1)	May be involved in sodium-dependent transport of neutral amino acids or oligopeptides or regulation of cation transport	Cluster B	XK mutations re associated with McLeod syndrome, a multisystem disorder with numerous abnormalities in the neuromuscular and hematopoietic systems. No known role for cancer

## ABCG2/BCRP1

ABCG2 first attracted wide attention from the scientific community when it was described as an ABC transporter expressed in a variety of stem cells and as a specific pump responsible for acquisition of a ‘side-population’ (SP) phenotype (Zhou et al., 2001). The AML SP contains leukemic stem cells (LSCs), and it can be detected in bone marrow and peripheral blood samples at diagnosis (Moshaver et al., 2008).

In earlier reports, as for other multidrug resistance (MDR) transporter proteins, ABCG2 had been implicated in the development of drug resistance (Benderra et al., 2004; Galimberti et al., 2004). However, more recent studies suggest that ABCG2 and MDR1 transporter-proteins have a limited role in drug resistance *in vivo* and *in vitro* (Svirnovski et al., 2009).

Nonetheless, high expression of ABCG2 mRNA is shown to be an independent prognostic factor for RR and DFS in adult AML. A correlation between the expression of the three major ABC pumps (ABCB1, ABCC1, and ABCG2) and *FLT3*-ITD and *MLL*-PTD (partial tandem duplication) in AML patient samples were also reported (Nasilowska-Adamska et al., 2014). Also, in adult *de novo* AML with normal karyotype, ABCG2-positive cases presented an increased risk of relapse, and ABCG2 overexpression inversely correlated with the duration of complete remissions (Damiani et al., 2006). Thus, the presence of up-regulated ABCG2 mRNA in the gene clusters that correlate with poor clinical outcome is well supported by other data. However, the upregulated message is not likely related to the drug resistance and clonal selection of cells overexpressing the pump, since tumor samples were obtained at the time of diagnosis (Valk et al., 2004), or from previously untreated individuals (Wilson et al., 2006).

### ATP1B2, Sodium/Potassium-Transporting ATPase Subunit Beta-2, AMOG

ATP1B2 is the non-catalytic component of the sodium/potassium-transporting ATPase (Na(+)-K(+)-ATPase), which catalyzes the hydrolysis of ATP coupled with the exchange of Na^+^ and K^+^ ions across the plasma membrane. The function of the beta-2 subunit is unclear, however, the beta-1 subunit could be important for targeting of the Na(+)-K(+)-ATPase to caveolae (Liu and Askari, 2006). Another function of ATP1B2 is in mediating cell adhesion on glia (AMOG), where it is highly expressed on the cell surface. AMOG is a heavily glycosylated protein that participates in cell adhesion in the CNS (Gloor et al., 1990). In glioblastoma-derived cells, expression of ATP1B2 down-modulates brain tumor-initiating cell invasion without significantly affecting cell proliferation (Sun et al., 2013).

In an effort to identify epigenetically silenced tumor suppressor genes (TSGs) in mouse mammary tumors, Demircan et al. (2009) cultured tumor cells with a demethylating drug. One of the TSGs identified was Atp1B2. In a panel of human breast tumors, authors observed that human ortholog ATP1B2 was aberrantly hypermethylated (Demircan et al., 2009). Epigenetic regulation of ATP1B2 expression was also reported in malignant gliomas (van den et al., 2006). The role of ATP1B2 in the pathogenesis of leukemia has yet to be investigated.

## CLCN3, H(+)/Cl(-) Exchange Transporter 3, ClC-3

ClC-3 belongs to the family of voltage-sensitive chloride channels^3^. ClC-3 is an antiporter that mediates exchange of protons against chloride ions, and participates in the acidification of endosomal compartments. It is expressed in tumors of different origin and reported to be specifically upregulated in glioma cells (Olsen et al., 2003). ClC-3 plays an important role in the regulation of cell cycle and cell migration, and specifically participates in the regulation of osmotic gradients and cell volume during cell-cycle progression (Habela et al., 2008; Mao et al., 2009; Cuddapah et al., 2012).

ClC-3 can be detected in late endosomal/lysosomal compartments, and through increased acidification of vesicles it increases cell resistance to the chemotherapeutic drug etoposide. This was achieved in the absence of a detectable expression of MDR transporters (Weylandt et al., 2007).

Also, ClC-3 is implicated in regulating apoptosis. In nasopharyngeal carcinoma cells, specific chloride channel blockers inhibited paclitaxel-induced apoptosis. At the same time, ClC-3-si-RNA knockdown experiments and other evidence suggests that ClC-3 represents a critical target for chemotherapeutic drug paclitaxel (Zhang et al., 2013). High expression of ClC-3 is reported to be a predictor of poor survival in lung adenocarcinoma, breast adenocarcinoma, and liver cancer, and this can be associated with the effect of the protein on tumor metastasis (Xu et al., 2014). A recently published review specifically addresses involvement of ClC-3 in cancer and explores its potential for therapy (Hong et al., 2015).

## CLIC2, Chloride Intracellular Channel Protein 2 (XAP121)

CLIC2 belongs to the family of chloride intracellular channel (CLIC) proteins^4^ that are implicated in membrane transport, cell division, secretion, and apoptosis (Ashley, 2003). These proteins possess an unusual feature: they can exist in a water soluble state and can insert into the membrane to form an anion channel (Ashley, 2003; Cromer et al., 2007; Jiang et al., 2014). CLIC2 is shown to interact with ryanodine receptor (RyR1), and to modulate calcium release from intracellular stores (Board et al., 2004; Meng et al., 2009). Some of the intracellular chloride channels from CLIC and other families are envisioned as proteins that “may contribute to a cancer phenotype” (Suh and Yuspa, 2005), however, no significant role for CLIC2 has been previously reported. Nonetheless, two related CLICs, CLIC1 and CLIC4, are overexpressed in cancer stem cells and envisioned as novel therapeutic targets (Peretti et al., 2014). Also, CLIC-like chloride channels are localized at the osteoclast ruﬄed border, and together with H^+^-ATPase, they participate in the acidification of the bone resorption compartment (Schlesinger et al., 1997; Supanchart and Kornak, 2008). Thus, CLIC2 represents a part of the plasma membrane machinery that actively triggers dissolution of the bone mineral matrix by lowering extracellular pH (Arnett, 2008), and leads to the dramatic changes in the niche microenvironment ionic composition.

## Solute Carrier (SLC) Proteins

Solute carrier is a large group of proteins that includes more than four dozen protein families and 395 transporter genes in human genome (Hediger et al., 2013)^5^. Both Cluster B in reference Wilson et al. (2006) and Clusters 7 and 8 in reference Valk et al. (2004) contained several members of this group (**Table 1**): SLC42 [Rhesus (Rh) glycoproteins, ammonium transporter family], SLC2A1 (glucose and/or fructose transporter GLUT1), SLC4A1 [bicarbonate transporter family anion exchanger (AE), member 1 (Diego blood group)], SLC6A8 (Sodium- and chloride-dependent creatine transporter 1), and SLC6A9 [sodium- and chloride-dependent glycine transporter 1 (GlyT1)]. In April–June 2013 *Molecular Aspects of Medicine* published a special issue (Volume 34, Issues 2–3, Pages 95–752) specifically dedicated to SLC transporters. This series of papers represents an excellent collection of in-depth reviews. Here, a summary of the most significant observations relevant in this context is presented.

## Rhesus Glycoproteins, SLC42 Transporters, RHAG, RHCE, RHD

Historically Rh glycoproteins were studied in human blood because of their immunogenicity and importance for gestation. The relationship between Rh protein expression and NH_3_/NH_4_^+^ was established in the late 1990s- early 2000s (Nakhoul and Lee, 2013). However, details which include stoichiometry, electrogenicity, and the exact nature of translocated substances are still unclear.

RhAG studies suggest that an electro-neutral transport of NH_4_^+^ is coupled with an antiport of a proton resulting in a net transport of NH_3_ (Westhoff et al., 2002). Other evidence suggests that RhAG directly participates in the transport of NH_4_^+^ and NH_3_ (Benjelloun et al., 2005). Transport of CO_2_ in a gaseous form or as HCO_3_^-^ is also plausible. It was also suggested that substrate specificity can be modulated by pH and other factors (Nakhoul and Lee, 2013). Thus, Rh proteins can play a significant role in the maintenance of acid-base balance as well as transport of NH_3_/methylamine/NH_4_(+)/CO_2_ (Van Kim et al., 2006; Nakhoul and Lee, 2013; Weiner and Verlander, 2014). No direct relationship to cancer or cancer cell metabolism has been previously reported. Diseases associated with Rh protein function include red blood cell-related disorders such as hemolytic disease of the newborn, auto-immune hemolytic anemia and others (Huang et al., 2000; Van Kim et al., 2006). Rh proteins are also related to chronic metabolic acidosis, impaired urinary NH_4_^+^ excretion in response to acid loads and other disorders where transport of ammonia and/or regulation of acid-base balance are critical (Seshadri et al., 2006; Biver et al., 2008). Rh protein family is also envisioned as a class of “ammonium sensor” proteins that may modulate diverse cellular functions in response to the changes in extracellular ammonium content (Van Kim et al., 2006).

## SLC2A1, GLUT1, Glucose Transporter Type 1

The protein encoded by human GLUT1 belongs to the sugar porter subfamily of the major facilitator superfamily (Henderson and Baldwin, 2013). It facilitates glucose diffusion, and is responsible for basal uptake of glucose. It also exhibits broad substrate specificity and is reported to transport a range of aldoses including both pentoses and hexoses (Thorens and Mueckler, 2010). GLUT1 is often overexpressed in a variety of tumors, suggesting that GLUT 1 is required for elevated glucose uptake in cancer (Amann and Hellerbrand, 2009; Ramani et al., 2013; Kaira et al., 2014), which can result from a specific metabolic phenomenon, aerobic glycolysis often described as the “Warburg effect”(Warburg, 1956b; Diaz-Ruiz et al., 2011).

In BCR-Abl+ B-cell acute lymphoblastic leukemia genetic deletion of Glut1 was sufficient to decrease glucose uptake and modify metabolic flux of glucose toward catabolism. *In vivo* Glut1 deletion resulted in suppression of disease progression. Authors concluded that B-cells mainly rely on Glut1 transport to maintain its metabolism, even in the presence of other glucose transporters (Liu et al., 2014). In AML patient samples high expression of GLUT1 was correlated with poor chemotherapy responsiveness. Authors concluded that GLUT1 could be a potential target to drug resistance reversal (Song et al., 2014). Also, GLUT1 represents one of the HIF-1α target genes (Majumder et al., 2004) that can be down-regulated by inhibitors of the mTOR signaling pathway in AML cells (Zeng et al., 2007), or specific HIF-1 inhibitors in B-cell CLL/lymphoma-2 (BCL2; Yonekura et al., 2013). Thus, an occurrence of up-regulated GLUT1 expression in the gene clusters that correlate with poor clinical outcome is not surprising given its well reported role in cancer in other studies.

## SLC4A1, Anion Exchanger, Member 1, Chloride-Bicarbonate Exchanger

SLC4A1, anion exchanger-1 (AE1), mediates the electroneutral Cl(-)/HCO(-)3 exchange across the plasma membrane, sulfate uptake, or proton and sulfate symport. It has been also suggested that the transport site of AE1 can also mediate the cation leak (Barneaud-Rocca et al., 2011). Other functions include association with the cytoskeleton through interaction with ankyrin and the actin/spectrin cytoskeleton, maintaining cell integrity and acid–base homeostasis and regulation of glycolysis (Walsh and Stewart, 2010; Wu et al., 2011).

In early 2000, it was noted that co-expression of AE1 cDNA together with Rh glycoprotein polypeptides (RHCE or RHD) increased the binding level of antibody specific to the endogenous Rh glycoproteins (Beckmann et al., 1998). Next, a macrocomplex consisting of AE1 and Rh proteins (Rh-associated glycoprotein, Rh polypeptides, glycophorin B, CD47 and LW) was described. Authors proposed that the macrocomplex represents an integrated CO_2_/O_2_/NO gas exchange unit (metabolon), where Rh proteins may act as “gas channels” that are located in close proximity to AE1 proteins providing H+ and HCO(-)3 movements necessary for efficient O_2_/CO_2_ exchange (Bruce et al., 2003). The model of the band 3 (AE1) macrocomplex has been modified to include glycolytic enzymes: aldolase, phosphofructokinase, and glyceraldehyde-3-phosphate dehydrogenase, and this association reduces enzymatic activity. An intricate model of AE1 macrcomplex regulation has been proposed (De Rosa et al., 2008).

No specific role for AE1 in hematological malignancies can be found. A series of reports describes the role of AE1 in gastric cancer. Expression of AE1 and cyclin-dependent kinase inhibitor tumor suppressor p16 has been studied in samples from patients with early and advanced stages of gastric cancer. The nuclear vs. cytoplasmic localization of p16 was dependent on the disease stage, with increased cytoplasmic localization in advanced cancer cells. P16 cytoplasmic expression correlated with AE1 expression, and both were associated with an absence of metastasis (Liu et al., 2009). Silencing of AE1 decreased cell proliferation. In a xenograft model experimental hypergastrinemia resulted in the down-regulation of AE1 and p16 expression and promoted growth inhibition (Tian et al., 2010). Another report revealed that AE1 expression in gastric carcinoma tumor samples strongly correlated with the onset and progression of cancer (Xu et al., 2009). In two mouse models of gastric cancer, the targeting of AE1 using siRNA resulted in down regulation of AE1 in gastric mucosa, decreased tumor growth, and lowered the rate of tumor detection. Authors concluded that AE1 silencing could provide a novel approach to treatment of gastric cancer (Suo et al., 2012).

An interesting study describes regulation of AE1 expression by the microRNA, miR-24 (Wu et al., 2010), which is critical for the development of normal hematopoietic progenitors, is highly expressed in primary AML, regulates hematopoietic cell survival (Nguyen et al., 2013), and is down-regulated during erythroid differentiation (Georgantas et al., 2007). AE1 protein demonstrates opposite dynamics of expression during differentiation. It is expressed only in a small fraction of early erythroid progenitors and it is up-regulated during erythroid differentiation (see Lehnert and Lodish, 1988) and (Table II in McAdams et al., 1998). Thus, this relationship suggests that miR-24 down regulation provides a regulatory mechanism for AE1 expression during differentiation (Wu et al., 2010).

In AML patient samples miR-24 expression has had no correlation with the mutations of genes *FLT3*-ITD, *NPM1*, C-*KIT*, *IDH1/IDH2*, *DNMT3A*, N/K-*Ras* and *C/EBPA*. However, miR-24 high expression was frequently detected in patients with *t*(8;21), with no significant correlation with OS or relapse-free survival (RFS; Yin et al., 2014). Since *t*(8;21) is often detected in patients with (French–American–British Classification) FAB AML-M2 subtype and represents one of the favorable abnormalities with one of highest complete remission rates after conventional chemotherapy (Nishii et al., 2003), (Reikvam et al., 2011), it is unlikely that Clusters 7 and 8 in reference Valk et al. (2004) and Cluster B in reference Wilson et al. (2006), which showed the worst DFS and OS possess high miR-24 expression. Though, no *t*(8;21) were detected in Cluster B (see Table 3 in Wilson et al., 2006), and in reference Valk et al. (2004), the *AML1-ETO* fusion gene was grouped within cluster 13.

## Solute Carrier (SLC6) Protein Family

Among genes up-regulated in poor outcome gene clusters, two belong to the family of SLC6 Na^+^/Cl^-^ dependent secondary active co-transporters (SLC6A8 and SLC6A9). The major functional role of SLC6 transporters is to mediate rapid uptake of small amino acid or amino acid-like substrates, neurotransmitters, amino acids, osmolytes and creatine, against very large concentration gradients (Pramod et al., 2013). SLC6 transporters exhibit different ion coupling stoichiometry, 1-3 Na^+^; 1-2 Cl^-^, to 1 substate molecule, or for some SLC6 members, a non-stoichiometric transport, as well as a Na^+^ leak current can be observed in the absence of substrate (Kristensen et al., 2011). Numerous, disease associations of SLC6 family members have been reported. Specifically, these are creatine deficiency syndrome, mental retardation, musculoskeletal disorders for SLC6A8, and schizophrenia and hypertension for SLC6A9 (Longo et al., 2011; Pramod et al., 2013). A specific role of SLC6A8 in the “import” of metabolic energy in colon cancer cells has been also described (Loo et al., 2015; Sullivan and Christofk, 2015).

## SLC6A8, Sodium- and Chloride-Dependent Creatine Transporter 1 (CT1; Creatine Transporter 1)

SLC6A8, creatine transporter 1 is one of the major creatine transporters in humans. It is associated with the plasma membrane and mediates an uptake of creatine in symport with sodium and chloride ions and, presumably, it provides creatine in cells where it cannot be synthesized (Nash et al., 1994; Sora et al., 1994; Pramod et al., 2013).

Creatine phosphate is one of the compounds that possess a high phosphoryl potential and therefore, it is required for the utilization of ATP-derived energy. Large levels of creatine are present in muscle, brain and heart, where rapid high energy production is essential for the physiology of the organ or tissue (Wyss and Kaddurah-Daouk, 2000; Longo et al., 2011). The fact that CT1 mRNA is up-regulated in gene clusters of poor-outcome AML hints at the possibility that these cells possess a high energy phenotype.

A recent report highlighted the role of SLC6A8 and creatine phosphate in the metastatic survival and colonization of colon cancer (Loo et al., 2015; Sullivan and Christofk, 2015). After a screen of 661 human miRNAs for their ability to suppress colonization of the liver by colon cancer cells, authors identified miR-551 and miR-483 as suppressors of colon cancer metastasis. Both miRNAs target brain-type creatine kinase (CKB) that is released by metastatic cells into the extracellular space where it catalyzes the formation of creatine phosphate from extracellular creatine and ATP. Next, creatine phosphate is taken up by cancer cells through SLC6A8 and is used to generate ATP and support survival of metastatic cells. Therapeutic viral delivery of two miRNAs decreased colon cancer metastasis. miRNA treatment significantly reduced expression of both (CKB and SLC6A8) proteins that were shown to be expressed at higher levels on metastatic cells as compared to primary tumors (Loo et al., 2015). Thus, SLC6A8 represents an essential part of a unique mechanism where creatine phosphate, a compound containing a high energy phosphate bond (∼P), is directly delivered into cancer cell, providing a substrate for transfer of its phosphoryl group to ADP and the generation ATP. Thus, the metabolic energy that is normally generated through intracellular processes that include glycolysis and oxidative phosphorylation can be directly “imported” into the cancer cell. Since SLC6A8 is up-regulated in samples obtained from poor-outcome AML patients, it is tempting to postulate the existence of a similar mechanism.

## SLC6A9, Sodium- and Chloride-Dependent Glycine Transporter 1 (GlyT-1)

SLC6A9, (glycine transporter 1) mediates rapid uptake of the amino acid glycine (Gly) in symport with sodium and chloride ions. Reported stoichiometry is 2 or 3 Na^+^ and 1 Cl^-^, per 1 Gly (Kristensen et al., 2011). It is responsible for rapid removal of the amino acid neurotransmitter Gly from the synaptic cleft and the termination of the neurotransmission signal (Lopez-Corcuera et al., 2001). In CNS GlyT1 is expressed on glial cells, as well as on neurons. It is localized on astrocytes and on pre-synaptic and post-synaptic membranes of glutamatergic synapses. GlyT1 transporter is reported to physically associate with the *N*-methyl-D-aspartate (NMDA) receptor (Raiteri and Raiteri, 2010).

SLC6A9 disease associations include schizophrenia (Tsai et al., 2006a,b; Deng et al., 2008), and hypertension (Ueno et al., 2009). GlyT gene deletion studies supported other data that indicate therapeutic potential of GlyT1 inhibitors for the treatment of psychiatric and neurological disorders (Aragon and Lopez-Corcuera, 2005). In cancer, GlyT1 transporters are implicated in regulation of pain, and GlyT inhibitors are envisioned as a novel class of drugs for neuropathic pain management (Dohi et al., 2009). For example, a recent report showed that oral or IV administration of GlyT1 inhibitor drastically improved pain-like behavior in a mouse femur bone cancer model. Authors concluded that GlyT inhibitors can provide a new path toward the treatment of bone cancer pain with morphine or alone (Motoyama et al., 2014). No specific role for GlyT1 has been previously reported for hematological malignancies.

## Selenium-Binding Protein 1 (56 kDa Selenium-Binding Protein), SBP56, SP56, SBP1

Because SBP1 protein can bind selenium, it has been suggested that SBP1 is implicated in the transport of the element (Chang et al., 1997). However, the exact SBP1 function remains unclear. Nevertheless, since it has been suggested that SBP1 protein may participate in intra-Golgi transport (Porat et al., 2000), it was included into this study.

During the last decade, numerous studies reported associations between SBP1 and cancer. In tumors of epithelial origin SBP1 is highly expressed in differentiated cells. A decrease in the expression of SBP1 detected at the level of mRNA or protein correlates with shorter DFS and OS in patient with colorectal tumors (Li et al., 2008). Expression of SBP1 was suppressed in gastric carcinoma; however, it was still high in precursor lesions. Reduced SBP1 expression was also associated with poor survival (Zhang et al., 2011a,b). In esophageal adenocarcinoma expression of SBP1 decreased significantly with disease progression. An overexpression of SBP1 elevated cisplatin sensitivity and promoted apoptosis in the presence of methylselenic acid. Thus, expression of SBP1 is envisioned as a predictor of the response to chemosensitization (Silvers et al., 2010).

Several studies point toward the role of epigenetic regulation of SBP1 expression. It was reported that SBP1 levels in colon cancer tissues are related to hypermethylation of the SBP1 promoter, and treatment with an epigenetic modifier that inhibits DNA methyltransferase activity resulted in the up-regulation of SBP1 mRNA and protein (Pohl et al., 2009). Epigenetic changes that included promoter hypermethylation were also reported in esophageal adenocarcinoma. Differential splicing and single nucleotide polymorphism also contributed to the regulation of SBP1 protein levels (Silvers et al., 2010). A recent review describes a specific role of SBP1 as a prognostic factor and tumor suppressor (Yang and Diamond, 2013).

Thus, it is not surprising that the SBP1 gene was detected in the gene cluster corresponding to the group of patients with poor-outcome adult AML. However, the fact that SBP1 was significantly up-regulated in both clusters [see positive sorting and assembly machinery (SAM) scores in supplemental data for (Valk et al., 2004)] poses a question about a physiological role of this protein in hematopoietic cells. No data about SBP1 in leukemia can be found.

## Mitochondrial Import Receptor Subunit TOM7, Translocase of Outer Membrane 7 kDa Subunit Homolog

TOM7 protein is an integral part of the translocase of the outer mitochondrial membrane (TOM complex) that provides an entry point for ∼99% of all precursor proteins in mitochondria. Since only 13 out of ∼1500 proteins in the mitochondrial proteome are encoded by the mitochondrial genome, the TOM complex represents an essential part for proper functioning of the organelle (Harbauer et al., 2014; Opalinska and Meisinger, 2014). The general import pore, where proteins are transported across the outer membrane (TOM), consists of multiple proteins designated: TOM22 and TOM40 stably associated central proteins, TOM20 and TOM70 protein precursor receptors, and the three small TOM proteins: TOM5, TOM6, and TOM7 (Rehling et al., 2001).

Earlier studies suggested that TOM7 may play a regulatory role in the assembly of the TOM complex. The deletion of TOM7 resulted in the stabilization of the complex, induced better association between TOM40 and TOM22, and reduced import of porin, also known as voltage-dependent anion-selective channel protein, one of the most abundant proteins in the mitochondrial outer membrane (Honlinger et al., 1996). The analysis of multiple single and double mutants of the three small Tom proteins in *Neurospora crassa* revealed that only mutants lacking both Tom6 and Tom7 proteins showed significantly altered phenotype. Single mutants lacking Tom7 or Tom6 exhibited drastically different abilities to import different proteins. Authors proposed that Tom7 and Tom 6 play diverse roles in the targeting/sorting of imported proteins into mitochondrial compartments (Sherman et al., 2005). However, as noted by Harbauer et al. (2014), it is important to remember that regulatory processes described in lower eukaryotes may differ between different organisms and cell types.

TOM7 is also implicated in the regulation of the SAM (complex) that participates in the transport of beta-barrel precursor proteins (Meisinger et al., 2006; Wideman et al., 2010). Tom7 was reported to disrupt the formation of the complete SAM and to promote segregation of the mitochondrial distribution and morphology protein Mdm10. Deletion of Tom7 stimulated formation of the complex between SAM and Mdm10. Authors concluded that Tom7 plays an important role in sorting and segregation of mitochondria outer membrane components (Meisinger et al., 2006). Also, Tom7 mediates Mdm10-dependent assembly of Tom40 complex by regulating its release from the SAM complex (Yamano et al., 2010).

Moreover, Tom7 plays an essential role in the biogenesis of mitochondria though modulation of two proteins: Tom40 and Tom22. At the early stage, it inhibits Tom40 complex formation by antagonizing Tom5 and Tom6. At the later stage, it induces the dissociation of the SAM-Mdm10 complex by sequestering unbound Mdm10, and thus delays the formation of Tom22:Tom40 complex (Becker et al., 2011). Therefore, Tom7 is one of the critical regulatory components responsible for the assembly and regulation of TOM and SAM complexes.

No direct association between cancer and TOM7 has been reported. However, numerous mitochondrial dysfunctions are associated with a large number of pathologies including cancer. Certain mitochondrial enzymes can act as tumor suppressors. Defects in isocitrate dehydrogenase, succinate dehydrogenase, and fumarate hydratase are implicated in gliomas (Nunnari and Suomalainen, 2012). Metabolic reprogramming, essential for rapid cell proliferation, requires synthesis of large quantities of amino acids, nucleotides and lipids and is directly related to aerobic glycolysis, also called the Warburg effect (Warburg, 1956a,b). Moreover, multiple components of mitochondrial import machinery are reported to be up-regulated in cancer and in some cases can be associated with poor prognosis. Certain components of the mitochondrial intermembrane space assembly complex were implicated in metabolic reprogramming during carcinogenesis (see Opalinska and Meisinger, 2014, and references therein). Thus, it was not surprising that one of the regulatory subunits of the TOM complex (TOM7) was found to be up-regulated in poor-outcome AML. The exact role of TOM7 in leukemogenesis requires further investigation.

## Membrane Transport Protein XK, Kell Complex 37 kDa Component, X-Linked Kx Blood Group

The protein encoded by XK X-linked Kx blood group gene possesses structural characteristics of membrane transport proteins. XK is considered a putative transporter of unknown function. However, based on protein similarity and experimental data it has been proposed that XK could be involved in sodium-dependent transport of neutral amino acids or oligopeptides or regulation of cation transport (Ho et al., 1994; Carbonnet et al., 1998; Rivera et al., 2013; also see Uniprot link in **Table 1**).

XK protein is expressed in a number of tissues including CNS and blood cells (Russo et al., 2000; Claperon et al., 2007), and mutations within XK protein are associated with McLeod syndrome, which is characterized as a multisystem disorder with numerous abnormalities in the neuromuscular and hematopoietic systems (Ho et al., 1994; Arnaud et al., 2009; Dubielecka et al., 2011; Zhu et al., 2014). In erythrocytes XK protein is associated with Kell protein, a zinc endopeptidase with endothelin-3-converting enzyme activity. It is proposed that the Kell/XK complex operate as a regulator of erythrocyte volume (Rivera et al., 2013). Kell is expressed on a broad range of haematopoietic cells including myeloid progenitors (Wagner et al., 2000). However, no known association with leukemia or other cancers has been reported in the literature.

**FIGURE 1:**
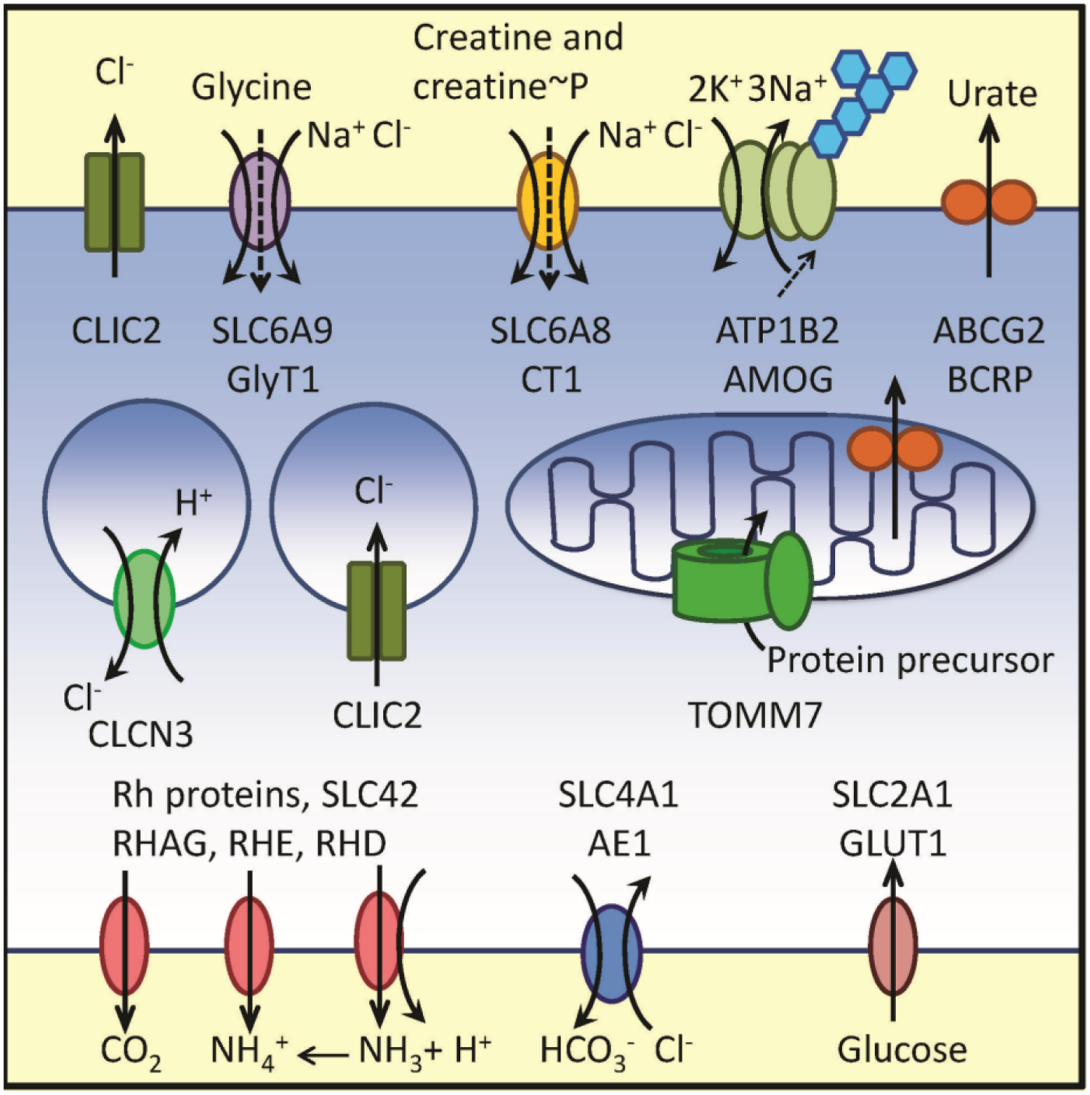
**Schematic representation of membrane transport-related proteins corresponding to up-regulated genes from Cluster B in reference Wilson et al. (2006) and Clusters 7 and 8 in reference Valk et al. (2004)**. Plasma membrane, endosomal compartment and a mitochondrion are shown. Transport directionality is indicated by arrows. Genes, protein names and transported substrates are shown. Sodium leak in SLC6 family is indicated using dashed arrows. Blue hexagons designate glycosylation of AMOG protein. Because physiological functions of SBP1 and XK remain unclear, these proteins are not shown in the figure. Notice a high number of proteins transporting chloride ion and nitrogen-containing substrates. For details see text and **Table 1**

Thus, the RNA message for multiple proteins involved in the membrane transport was reported to be up-regulated in samples obtained from adult AML patients with poor outcome. The data are graphically summarized in **Figure 1**

## Transporter Up-Regulation, What Does It Mean?

At first glance, based on the nature of channel/transporter substrates, this group of proteins can be divided into two: transporters involved in transport of nitrogen containing compounds and products of nitrogen metabolism (ABCG2, SLC6A9, SLC6A8, SLC42A1, RHCE, RHD), and channels/transporters involved in regulation of acid-base balance and inorganic ion transport (CLIC2, ATP1B2, CLCN3, SLC4A1, SLA42 proteins, XK). Glucose transporter GLUT1, BP1 and TOM7 are not directly related to either group. Analysis of the existing literature suggested four major ideas regarding a potential relationship between overexpressed genes encoding transport proteins and possible physiological features of these samples. These lines of reasoning included: (a) cell metabolic specificity related to the Warburg effect, overproduction of lactate and carbonic acids, and an alteration of membrane transport as a result of intracellular pH (pHi) regulation; (b) an unusually high number of proteins implicated in the transport of chloride; (c) metabolic alterations resulting from SLC6A8 overexpression and a possibility of increased uptake of creatine phosphate; (d) a loss of integrin dependent adhesion and AML cell mis-homing triggered by a massive modification of the ion transport within the niche microenvironment. None of these ideas are mutually exclusive. Because patient samples were obtained at the time of diagnosis or from previously untreated individuals, it is safe to assume that no drug-induced clonal selection contributed to the expression profiles.

## “Warburg Effect,” pHi and Membrane Transport

The last decade has been manifested by an increase in the number of papers dedicated to the role of energy consumption, glycolytic metabolism, pH regulation, and ion transport in cancer. Numerous excellent reviews of the topic exist (Doherty and Cleveland, 2013; Parks et al., 2013; Reshkin et al., 2014; Swietach et al., 2014). The general model of the pHi regulation in cancer is being developed, and it is generally accepted that tumor cells require a “coordination of multiple proteins” to effectively regulate pHi in the highly acidic environment of the tumor niche (Parks et al., 2013). Therefore, only several relevant genes and proteins whose impact has been well documented previously are described below.

The up-regulation of GLUT1 transporter, reported in multiple cancers, is usually attributed to aerobic glycolysis (Warburg effect; Diaz-Ruiz et al., 2011). The expression of GLUT1 is under the control of hypoxia-inducible factors (HIFs)-1 and -2, suggesting a regulatory mechanism directly triggered by hypoxic conditions created and maintained in rapidly growing tumors (Chan et al., 2011). The switch to aerobic glycolysis with increased lactate generation can be also modulated through PI3K/AKT signaling that contributes to the cell surface expression of GLUT transporters and activation of multiple glycolytic enzymes (Doherty and Cleveland, 2013). Thus, GLUT1 without being directly involved in a regulation of acid-base balance in cancer cells provides a large influx of glucose that serves as a major source of lactate. The other metabolic substrate for aerobic glycolysis is glutamine, which is transported through SLC1A5 (sodium-dependent amino acids transporter, ASCT2; Doherty and Cleveland, 2013) that has not been up-regulated in the studied clusters [Cluster B in reference Wilson et al. (2006) and Clusters 7 and 8 in reference Valk et al. (2004)]. It is worth noting that SLC16A1 (proton-coupled monocarboxylate transporter 1) and SLC16A3 (proton-linked monocarboxylate transporter 4), two transporters implicated in the shuttling of lactate across the cancer cell plasma membrane, were also not up-regulated.

The other gene directly implicated in the regulation of pHi is SLC4A1, an AE1 that mediates the electroneutral Cl(-)/HCO(-) 3 exchange across the plasma membrane. AE1 is an integral part of a membrane complex involved in HCO(-)3 transport and regulation termed “transport metabolon.” According to the model, the co-localization of two major components of the metabolon, carbonic anhydrase II and AE1 facilitate rapid carbon dioxide transport across the lipid bilayer (Sterling et al., 2001). The fact that AE1 is described to form a macrocomplex (called band 3 macrocomplex) with Rh proteins, also up-regulated in studied clusters (**Table 1**), suggests a specific functional relationship between these proteins in AML cells (Bruce et al., 2003; De Rosa et al., 2008). Because the last complex is envisioned as CO_2_/O_2_/NO gas exchange unit, this may point toward a possible role of nitric oxide in AML pathogenesis.

## Chloride Transport

One surprising feature that can be noticed after **Figure 1** examination is a high incidence of channels and transporters implicated in the transport of chloride ions. A normal serum concentration of chloride ion is much larger than a cytosolic concentration. This gradient can be a part of the force driving an uphill symport of several organic metabolites, such as amino acids, amino acid-like substrates and creatine or creatine phosphate (see refs. for SLC6 family above). If genes that are involved in the membrane transport of a chloride ion are up-regulated in a poor outcome AML, it might point to the possibility of a specific role of chloride ion transport in leukemia. Several studies describe targeting chloride transport in cancer. Chloride channels (CLICs specifically) are implicated in tumor development. Together with ClC-3, CLICs are perceived as potential therapeutic targets in solid tumors, see (Peretti et al., 2014) and references therein. However, additional studies are needed to better understand the role of chloride channels cancer pathogenesis.

The other line of research is related to the studies of small molecules capable of modifying chloride transport. Prodigiosins, a family of tripyrrolic molecules of natural origin exhibit anticancer activity. One molecular mechanism proposed to explain prodigiosin anticancer activity is related to its ability to stimulate a symport of protons with chloride ions, and thus, to uncouple proton translocation machinery (Sato et al., 1998). A synthesis of a series of structurally diverse prodigiosin analogs revealed that the rate of ion translocation, rather than anion binding ability, correlates with the anticancer activity (Sessler et al., 2005). This line of research resulted in the synthesis of novel compounds capable of facilitating passive chloride influx followed by sodium entry via sodium channels, which stimulated formation of reactive oxygen species, a release of cytochrome c and apoptosis via caspase activation (Ko et al., 2014). Synthetic chloride ion transporters (or carriers) that dramatically alter chloride flux across the cell membrane may represent an interesting option for targeting cells that significantly overexpress chloride ion transporters.

Nevertheless, the majority of studies of proton dynamics, lactate metabolism and ion transport were conducted in model systems representing solid tumors that include colon, breast, brain, lung, and prostate cancer. The role of transport mechanisms in leukemia is less studied. Our current data highlight a possible role of transporters that can be related to pHi stabilization or chloride ion transport in adult AML. It is possible that despite a significant heterogeneity of AML samples, certain subsets can be identified and used for future diagnostic or therapeutic application.

## Metabolic Alterations and SLC6A8 Overexpression

Another exciting hypothesis that originated from the analysis of studied clusters is related to the overexpression of SLC6A8, creatine transporter 1. Recently, Loo et al. (2015) described a model, where a malignant cell can acquire metabolic energy thorough the uptake of creatine phosphate. First, creatine phosphate is synthesized outside of a cancer cell from creatine and ATP, provided by an adjacent non-malignant cell. Next, creatine phosphate is taken up by a cancer cell via SLC6A8. Finally, the high-energy phosphate group from creatine phosphate is utilized inside of a cancer cell to generate ATP. In this case, the malignant cell will have no need for the production of large quantities of “its own” domestically produced ATP. Using this mechanism metabolic energy can be directly “imported” in the form of creatine phosphate. Consequently, the major cellular metabolic pathways can be reprogrammed for the synthesis of nucleotides and amino acids to sustain rapid cell proliferation.

However, since for every phosphocreatine molecule only one high energy phosphate is transferred, the process requires a “shuttle” mechanism, where phosphocreatine enters the cancer cell through SLC6A8 and after transfer of a high-energy bond, creatine or creatine metabolites are returned back to the extracellular space for the next round of creatine phosphorylation. This proposed mechanism appears to be different from a well described “phosphocreatine shuttle” system, where a high-energy phosphate, present in ATP, is transferred intracellularly from mitochondria to the cytosol (Bessman and Geiger, 1981). Also, rather than being eﬄuxed from a cancer cell, intracellular creatine is likely metabolized and metabolic products are removed using transporters located in the plasma membrane. This might explain an unusual up-regulation of proteins implicated in the transport of nitrogen containing compounds and products of nitrogen metabolism (**Figure 1**).

## Membrane Transport and Niche Biology

Another idea is related to the “restraining effect” within the tumor microenvironment that under normal conditions suppresses tumor development (Bissell and Hines, 2011). An interaction between adhesion molecules (beta1-integrin, for example) expressed on tumor cells, and components of the niche is envisioned as one of the underlying molecular mechanisms of this “restraining effect” (Correia and Bissell, 2012). Binding of ligands to beta1-integrins [and specifically to very late antigen-4 (VLA-4, CD49d/CD29), the major integrin responsible for the homing retention of early hematopoietic progenitors (Rettig et al., 2012)] is regulated through a series of conformational changes (Chigaev and Sklar, 2012) which are extremely sensitive to ionic conditions and the redox microenvironment (Chigaev et al., 2003, 2004; Liu et al., 2008). Therefore, it is possible that the overexpression of proteins, implicated in regulation of acid-base balance and acidification of the extracellular milieu, will dramatically alter adhesive interactions between AML cells and bone marrow niche components. We envision that the loss of this critical adhesive interaction will result in a disruption of the normal niche microenvironment and the creation of an abnormal cellular ecosystem. The loss of proper cell homing within the niche can also contribute to altered epigenetic regulation. Radical modification of the bone marrow microenvironment by leukemic cells has been reported previously (Colmone et al., 2008).

## Conclusion

Targeting transporters and channels in cancer is presently envisioned as novel and very promising area of anticancer therapy (Arcangeli et al., 2009; Oosterwijk and Gillies, 2014). A successful use of drugs targeting ion channels for treatment of multiple diseases unrelated to cancer indicates that these drugs can be well tolerated and safely used over a long time period. Unfortunately, the majority of current studies of membrane proteins in cancer are largely limited to solid tumors. Roles of transport proteins as therapeutic targets and pathogenesis of leukemia remain largely unexplored.

## Conflict of Interest Statement

The author declares that the research was conducted in the absence of any commercial or financial relationships that could be construed as a potential conflict of interest.

## Footnotes

1 http://www.uniprot.org/uniprot/

2 http://www.ebi.ac.uk/QuickGO/

3 http://www.genenames.org/genefamilies/CLCNS\#CLCN

4 http://www.genenames.org/genefamilies/CLCNS\#CLIC

5 http://www.genenames.org/genefamilies/SLC
